# Massive haemorrhagic complications of ruptured pulmonary arteriovenous malformations: outcomes from a 12 years’ retrospective study

**DOI:** 10.1186/s12890-021-01604-5

**Published:** 2021-07-13

**Authors:** Xu Ma, Bing Jie, Dong Yu, Ling-Ling Li, Sen Jiang

**Affiliations:** Department of Radiology, Shanghai Pulmonary Hospital, Tongji University School of Medicine, 507 Zhengmin Road, Shanghai, 200433 China

**Keywords:** Pulmonary arteriovenous malformation, Haemoptysis, Haemothorax, Computed tomography angiography, Embolisation

## Abstract

**Background:**

The life-threatening haemorrhagic complications of pulmonary arteriovenous malformations (PAVMs) are extremely rare, and only described in isolated cases. This study was designed to comprehensively investigate management of ruptured PAVMs.

**Methods:**

We retrospectively assessed clinical and imaging data of ruptured PAVMs to summarize incidence, clinical characteristics, and outcomes following embolisation between January 2008 and January 2021.

**Results:**

Eighteen of 406 (4.4%) patients with PAVMs developed haemorrhagic complications. Twelve of 18 patients were clinically diagnosed with hereditary haemorrhagic telangiectasia (HHT). Haemorrhagic complications occurred with no clear trigger in all cases. Eight of 18 patients (44.4%) were initially misdiagnosed or had undergone early ineffective treatment. 28 lesions were detected, with 89.3% of them located in peripheral lung. Computed tomography angiography (CTA) showed indirect signs to indicate ruptured PAVMs in all cases. Lower haemoglobin concentrations were associated with the diameter of afferent arteries in the ruptured lesions. Successful embolotherapy was achieved in all cases. After embolotherapy, arterial oxygen saturation improved and bleeding was controlled (*P* < 0.05). The mean follow-up time was 3.2 ± 2.5 years (range, 7 months to 10 years).

**Conclusions:**

Life threatening haemorrhagic complications of PAVMs are rare, they usually occur without a trigger and can be easily misdiagnosed. HHT and larger size of afferent arteries are major risk factors of these complications. CTA is a useful tool for diagnosis and therapeutic guidance for ruptured PAVMs. Embolotherapy is an effective therapy for this life-threatening complication.

## Background

Pulmonary arteriovenous malformations (PAVMs) are abnormal direct communications between the pulmonary arteries and veins through a thin-walled aneurysm [1]. As a result, blood bypasses the pulmonary capillary bed with resultant intrapulmonary right-to-left shunt [2, 3]. Most PAVMs are closely associated with hereditary haemorrhagic telangiectasia (HHT), an autosomal dominant genetic disorder [3–6]. The prevalence of PAVMs is estimated at 1 in 2,630 individuals [7–10]. Multiple PAVMs are more frequent in patients with HHT [11]. Women are more often affected than men, and single lesions are more common than bilateral or multiple lesions [12–14].

Most PAVMs cause no symptoms [15] and few cause hypoxemia, cyanosis [6, 16]. Other complications such as stroke and brain abscess also occur in a few patients, potentially due to the deficient filtration function in the pulmonary capillary bed [6, 17–19].

In the fully developed PAVM, the sac of the PAVM is markedly dilated and convoluted, and has excessive layers of smooth muscle without elastic fibers. The vessel wall of the sac is fragile and cannot vasoconstrict. Once the sac of PAVM ruptures, high blood flow from the afferent artery can result in massive haemorrhage. Although haemoptysis and haemothorax rarely occur following rupture of PAVMs, they are life-threatening complications if they are not diagnosed and treated in a timely manner [8, 20–23].

Endovascular management of PAVMs has been the first-line treatment in lieu of surgery over the last few decades [6, 24, 25]. Treatment of ruptured PAVMs has only been described in isolated case reports [26]. Accordingly, the present study was performed to retrospectively evaluate cases of ruptured PAVMs treated at our institution and to highlight their incidence, clinical characters, management, and outcomes.

## Materials and methods

### Characteristics of patients and PAVMs

This study involved 406 patients who were diagnosed with PAVMs based on multi-detector computed tomography (CT) or multi-detector CT angiography (CTA) in our hospital between January 2008 and January 2021. Among these 406 subjects, we analyzed patients who presented to the emergency department with haemoptysis or haemothorax and received embolotherapy. A clinical diagnosis of HHT was made according to the Curacao criteria: 1) spontaneous, recurrent epistaxis; 2) multiple telangiectasias, especially in the superficial mucosa; 3) visceral lesions such as in gastrointestinal mucosa, liver, and brain; 4) first-degree relatives with HHT. Clinical diagnosis of HHT was confirmed when at least two of the criteria were met (two for possible HHT, three or more for definite HHT) [21].

PAVMs were classified as either simple or complex according to their imaging characteristics (on CTA or pulmonary angiography) [3, 6, 9, 27]. PAVMs with only one segmental afferent artery were classified as simple PAVMs. PAVMs with two or more segmental afferent arteries were classified as complex PAVMs. PAVMs were further grouped into three groups according to their location and number: solitary PAVMs, unilateral multiple PAVMs, and bilateral multiple PAVMs.

### Treatment process

Emergency transvascular embolisation was performed by three experienced interventional radiologists with 10, 12, and 15 years of experience, respectively. Patients underwent pre-procedural emergency CTA (slice thickness: 0.625 mm; slice gap: 0.625 mm). The diameter of the afferent arteries was measured for all PAVMs. Embolotherapy was performed from a transfemoral vein approach with placement of embolisation coils (Cook Medical, Bloomington, IN, USA) or plugs (AGA Medical, Plymouth, MN, USA) in the distal aspect of all suitable PAVMs (with feeding arteries ≥ 3 mm in diameter) [3, 6, 28–32].

Partial pressure of oxygen (PaO2), arterial oxygen saturation (SaO2), haemoglobin concentration, leukocyte count, blood coagulation function, electrocardiograph (ECG) and clinical symptoms were recorded before and 2 days after the embolotherapy. Oxygen threrapy (oxygen by nasal cannula at 3L/minute) was administered after embolisation. Blood transfusion or closed thoracic drainage were performed when clinically indicated.

### Follow-up

Embolotherapy was considered successful if pulmonary haemorrhage was absent after treatment. All patients were followed up. CTA was repeated in the first month after treatment, and a chest CT scan was performed at the next follow-up if the CTA at the first follow-up showed no evidence of PAVM recurrence. Follow-up was terminated if symptoms requiring hospital admission recurred or the patient died.

### Statistical analysis

All results are expressed as mean ± standard deviation, and the statistical analysis was performed using SPSS version 19.0 (IBM Corp., Armonk, NY, USA). The survival time was analyzed with Kaplan–Meier curves and the log-rank test. A paired-sample t-test was applied to assess the statistical significance of differences. The impact of the afferent arterial diameter on the haemoglobin concentration was examined by simple linear regression and variable correlation scatter plots. For all analyses, a *P*-value of < 0.05 was considered statistically significant.

## Results

The study population consisted of 18 patients with ruptured PAVMs out of 406 patients diagnosed with PAVMs from January 2008 to January 2021.

### Clinical features

The patients’ basic characteristics are shown in Table 1. Thirteen patients had haemoptysis and five had haemothorax. PAVM rupture occurred with no clear trigger in all cases. The incidence of these haemorrhagic complications caused by acute rupture of PAVMs was 4.4% (18/406). Eleven (61.1%) of 18 patients were female (eight with haemoptysis and three with haemothorax). A clinical diagnosis of definite or possible HHT was made in 12 patients. One patient in the haemoptysis group had hepatic cirrhosis. This was considered to be the only case of acquired PAVM in our study. Before treatment, all patients were mildly hypoxemic and mildly anemic. The white cell blood count was within normal limits (Table 1). Twelve of the 18 patients (66.7%) didn’t have any other underlying diseases except for HHT.

**Table 1:** Baseline characteristics of patients with acute ruptured PAVMs

	Haemoptysis	Haemothorax
Subjects n	13	5
Age in years (range)	48.7 ± 15.8 (17–67)	53.4 ± 23.7 (18–72)
Female/Male	8/5	3/2
HHT (F/M)		
Definite	7 (4/3)	3 (2/1)
Possible	2 (2/0)	0
SaO2 (%)	95.2 ± 2.8	90.7 ± 6.9
PaO2 (mmHg)	76.3 ± 13.0	61.3 ± 10.2
Haemoglobin (g/L)	102.4 ± 24.6	100.7 ± 10.9
Leukocyte (10^9/L)	8.0 ± 3.5	5.3 ± 1.2
Concomitant diseases		
Tuberculosis	1	0
Bronchiectasis	2	1
Chronic bronchitis	0	2
None	10	2

Data are presented as n or mean ± standard deviation. *PAVM* pulmonary arteriovenous malformation, *HHT* hereditary haemorrhagic telangiectasia, *PaO2* partial pressure of oxygen, *SaO2* arterial oxygen saturation

### Characteristics of PAVMs

Six lesions were detected in the haemothorax group: solitary lesions in four patients and unilateral multiple lesions in one patient. All lesions were the simple type (Table 2). Twenty-two lesions were detected in the haemoptysis group: a solitary PAVM in seven patients, unilateral multiple PAVMs in one patient, and bilateral multiple PAVMs in five patients. Seventeen of the 22 lesions were the simple type, and the rest were the complex.

**Table 2:** Characteristics of detected PAVMs

Patient	Multiplicity (Number)	HHT	Type	Location distribution	Largest diameter of afferent artery (mm)
		(N/P/D)	(S/C)		
Haemothorax					
1	Unilateral multiple (2)	D	S	RLL A6 subpleural	6.4
			S	RLL A7 subpleural^#^	5.8
2	Solitary	D	S	LLL A9 subpleural^#^	5.6
3	Solitary	N	S	RLL A10 subpleural^#^	4.5
4	Solitary	D	S	LLL A9 subpleural^#^	6.1
5	Solitary	N	S	LLL A9 subpleural^#^	8.2
Haemoptysis					
1	Solitary	N	C	RLL A9 + 10 subpleural^#^	4.4
2	Solitary	N	S	RLLA9 subpleural^#^	3.2
3	Solitary	D	C	LUL A1 + 2 subpleural^#^	5.3
4	Bilateral multiple (4)	D	S	RML A4 + 5 subpleural	4.4
			S	RLL A10 outer 1/3^#^	3.7
			C	LUL A1 + 2 outer 1/3	5.9
			S	LLL A7 + 8 inner	3
5	Solitary	N	S	LUL A5 subpleural^#^	3.1
6	Bilateral multiple (2)	D	S	RLL A9 subpleural	3.9
			S	LLL A10 outer 1/3^#^	6.4
7	Solitary	N	S	RML A5 subpleural^#^	4.2
8	Unilateral multiple (3)	P	S	LUL A3 subpleural	3.2
			S	LUL A5 inner	3
			S	LLL A7 + 8 subpleural^#^	4.9
9	Solitary	D	S	RML A5 outer 1/3^#^	5
10	Unilateral multiple (2)	D	S	RML A5 subpleural	3.1
			S	RLL A9 outer 1/3^#^	4.2
11	Bilateral multiple (2)	D	C	RUL A3 inner	6.5
			S	LUL A5 subpleural^#^	5.7
12	Bilateral multiple (2)	D	C	RLL A10 outer 1/3	5.2
			S	LLL A10 subpleural^#^	7.1
13	Solitary	P	S	LLL A6 subpleural^#^	3.8

*PAVM* pulmonary arteriovenous malformation, *HHT* hereditary haemorrhagic telangiectasia, *N* none, *P* possible HHT, *D* definite HHT, *S* simple type, *C* complex type, *RUL* right upper lobe, *RML* right middle lobe, *RLL* right lower lobe, *LUL* left upper lobe, *LLL* left lower lobe, *A* artery of pulmonary segment; ^#^ruptured lesions

**Fig. 1:**
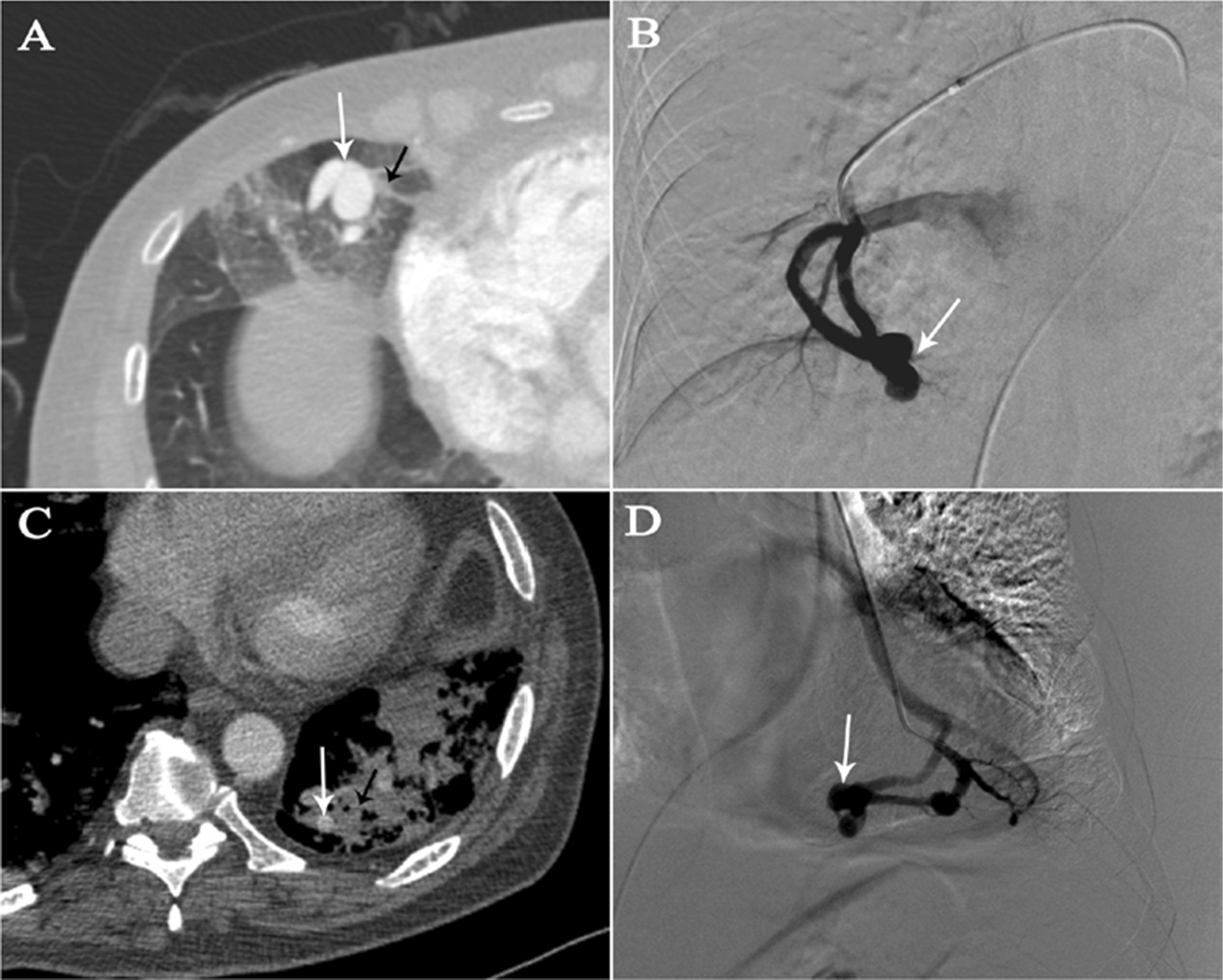
Ruptured PAVMs were in close proximity to pulmonary consolidations or ground-glass opacity in haemoptysis group. **A**, **B** a 67-year-old woman with massive haemoptysis. The white arrow points to the ruptured PAVMs and the black arrow indicates the adjacent ground-glass opacity. **C**, **D** a 60-year-old man with life-threatening haemoptysis. One ruptured PAVM (white arrow) was surrounded by consolidation (black arrow)

**Fig. 2:**
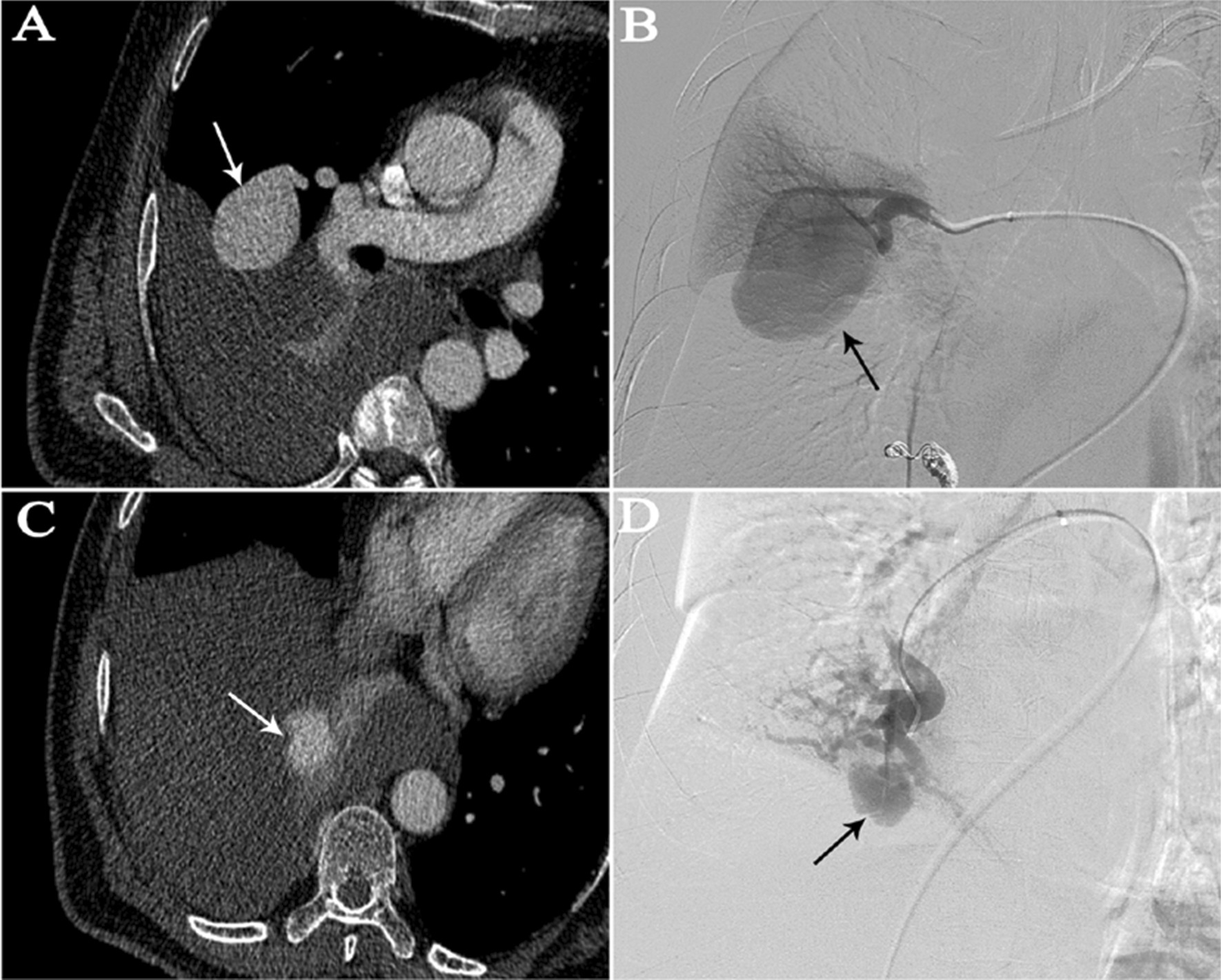
Unilateral multiple PAVMs in a 52-year-old man with haemothorax. **A**, **B** one smooth PAVM located in the interlobar pleural area was not considered to be ruptured based on its appearance on multi-detector CTA and pulmonary angiography (arrows). **C** the ruptured lesion located in the subpleural area manifested as an “anomalous bulge” on CTA (arrow). **D** this characteristic presented as the “double shadow sign” on angiography, as indicated by the black arrow

**Fig. 3:**
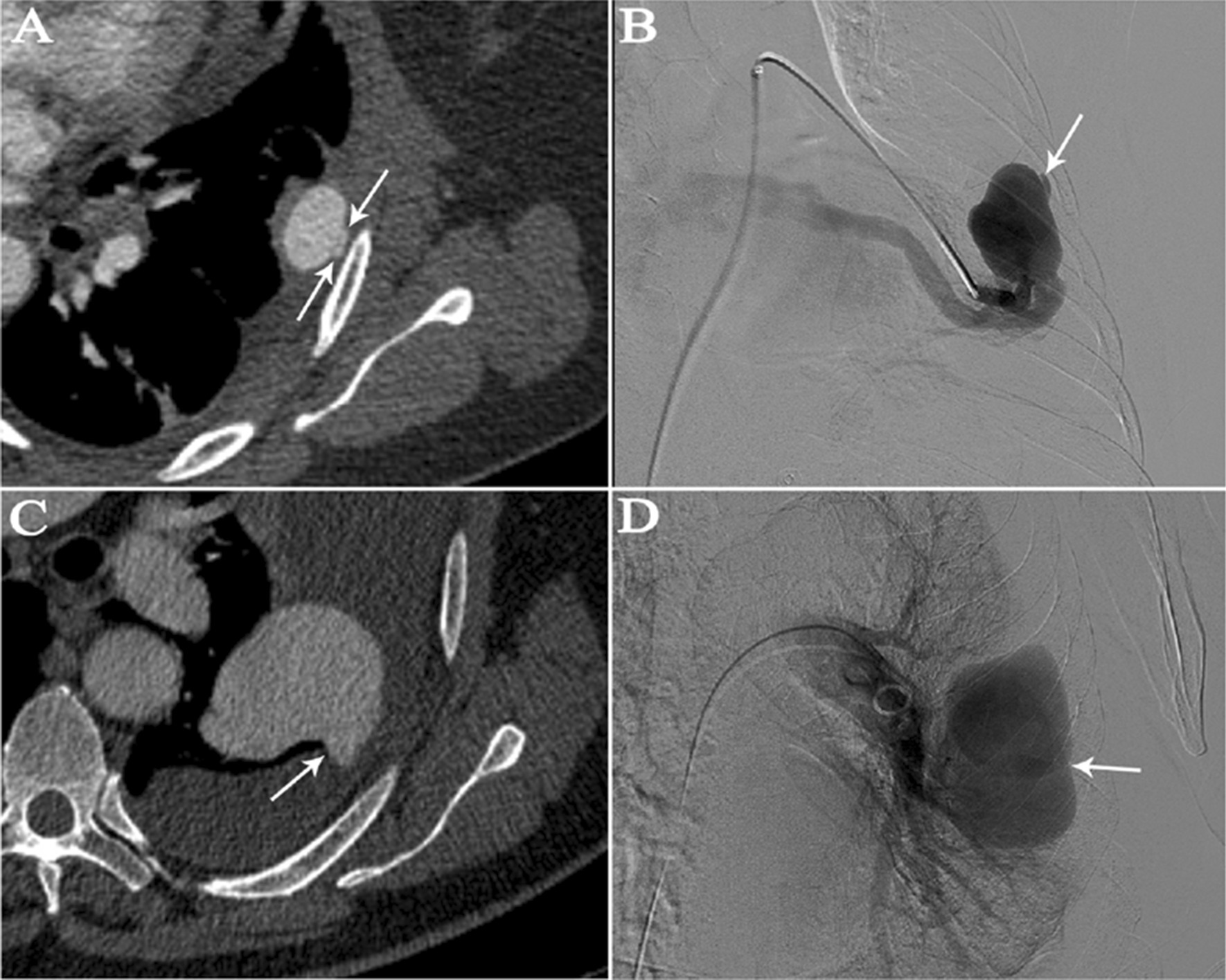
**A**, **B** a 47-year-old woman with haemothorax; **C**, **D** a 72-year-old man with haemothorax. **A**, **C** “Anomalous bulge” sign on CTA: vessel wall of ruptured PAVM and (supposedly) pulled towards the pleura by the negative intrathoracic pressure (arrows). **B**, **D** the overlap of “anomalous bulge” and the adjacent PAVM’s sac gives rise to the “double shadow sign” on the 2D-angiography (arrows)

**Fig. 4:**
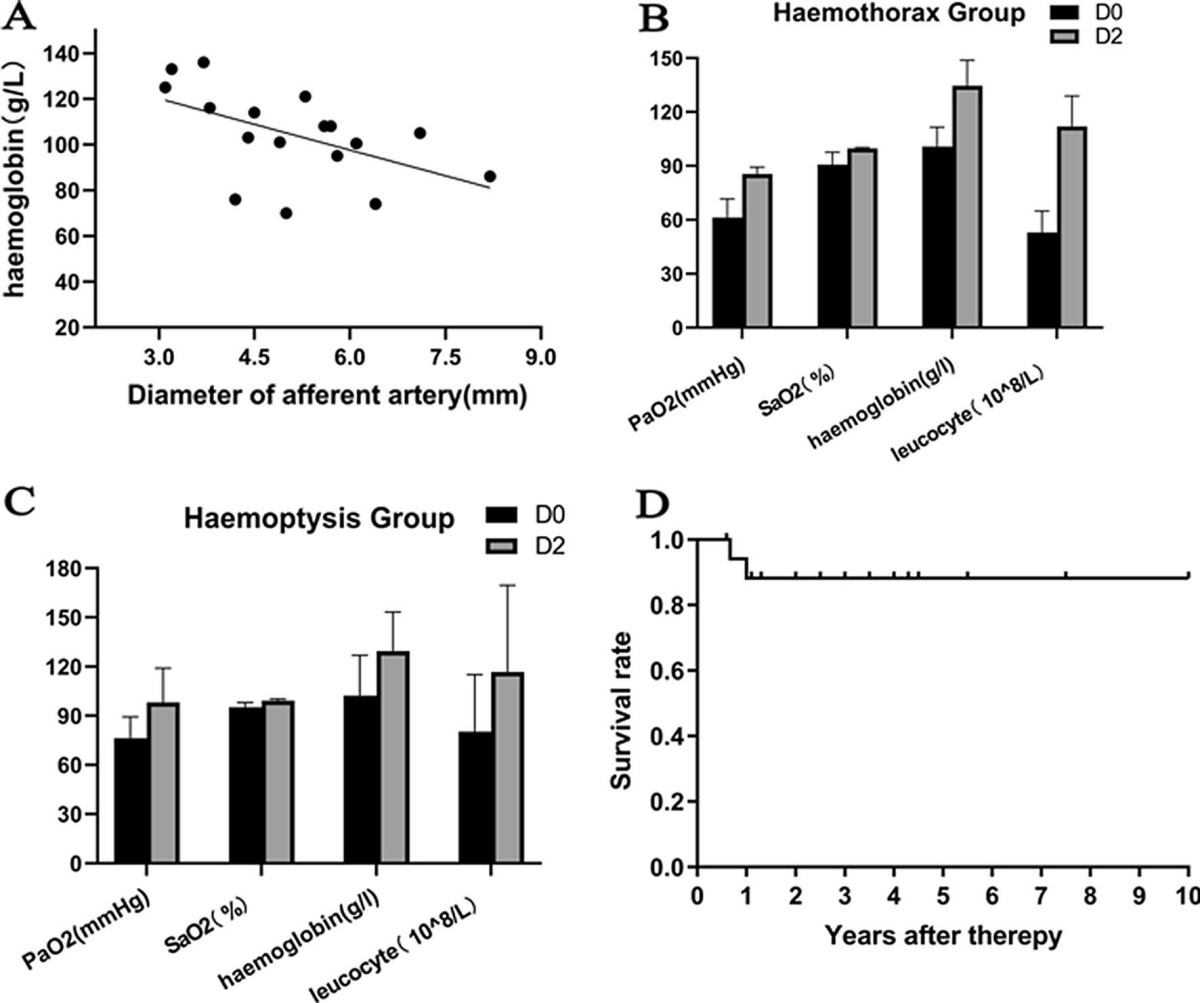
**A** lower levels of haemoglobin concentration were linearly associated with the diameter of the afferent arteries in the ruptured lesions (*P* = 0.029); **B**, **C** in the haemoptysis and haemothorax groups, PaO2, SaO2, haemoglobin concentration, and leukocyte count increased after therapy (*P* = 0.004, *P* < 0.001, *P* = 0.009, and *P* = 0.048, respectively in the haemoptysis group) (*P* < 0.001, *P* < 0.02, *P* = 0.003, and *P* < 0.001, respectively in the haemothorax group); D0 = the day when embolotherapy was performed; D2 = two days after embolotherapy; PaO2 = partial pressure of oxygen; SaO2 = arterial oxygen saturation; **D** Kaplan–Meier analysis for overall survival. The mean survival time was 3.2 ± 2.5 years, ranging from 7 months to 10 years

In the haemothorax group, all lesions were located in the subpleural area. In the haemoptysis group, 19 of the 22 (86.4%) PAVMs were peripheral, and 15 of 22 (68.2%) were located at the middle or lower lobes (Table 2). In the haemoptysis group, ruptured PAVMs were in close proximity to pulmonary consolidations or ground-glass opacities (Fig. 1). On CTA, the vessel wall of the ruptured PAVM’s sac in the haemothorax group was pulled towards the pleura (probably as a result of the negative intrathoracic pressure) giving rise to the so-called “anomalous bulge” on CTA (Fig. 2). During 2D-imaging angiography, the overlap of “anomalous bulge” and the adjacent sac gave rise to the so-called “double shadow sign” (Fig. 2). We observed this finding in all five ruptured lesions (Fig. 3). The mean diameter of afferent arteries in the ruptured lesions was 5.1 ± 1.4 mm (range, 3.1–8.2 mm). The diameter of the afferent arteries of the ruptured PAVMs was related with severity of anemia (*P* = 0.029) (Fig. 4A).

### Treatment process

**Fig. 5:**
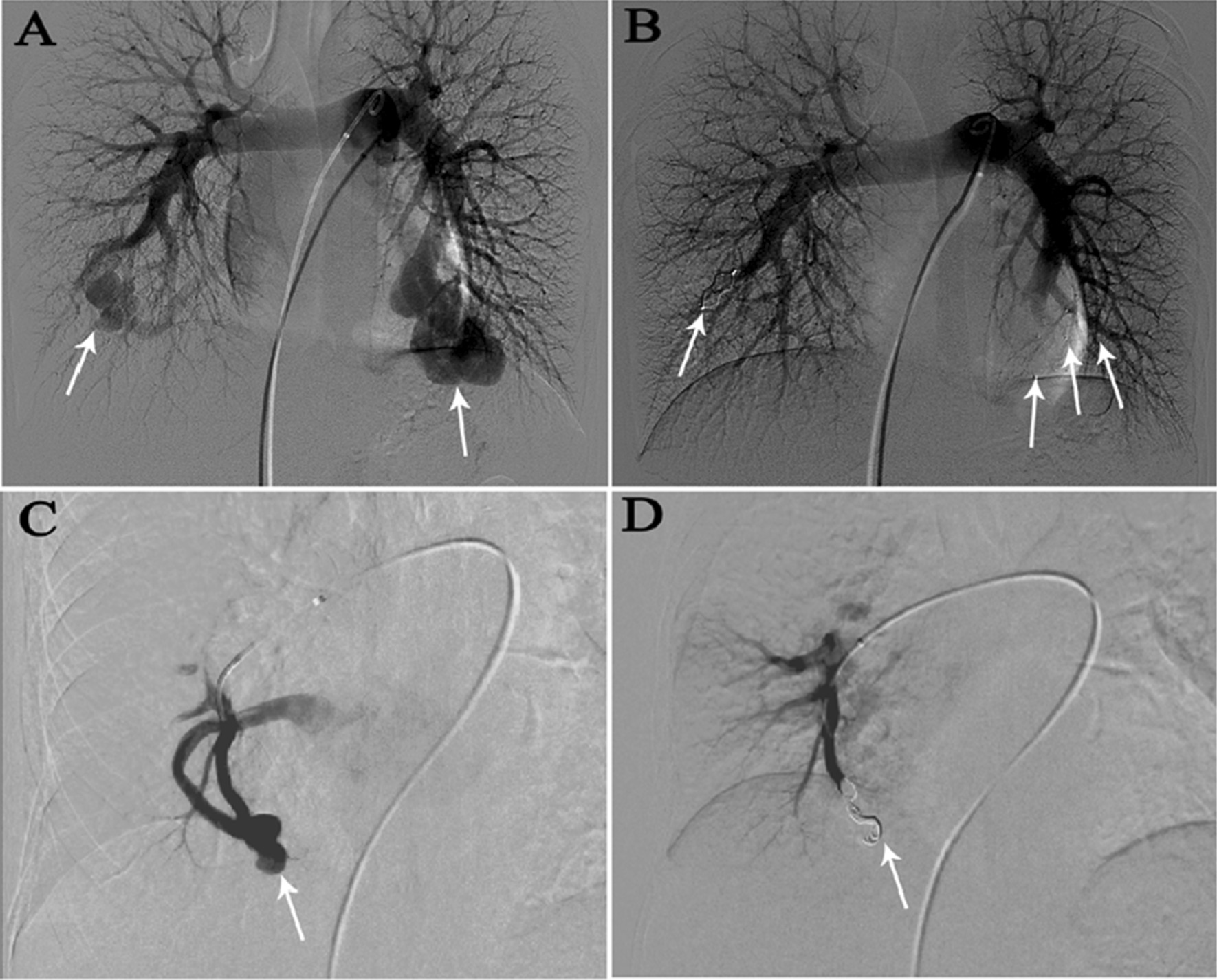
**A**, **B** pulmonary angiogram of a 17-year-old man with haemoptysis; **A** bilateral multiple PAVMs were detected in the lower lobe (arrows). **B** embolisation was performed with four plugs (arrows). **C**, **D** pulmonary angiogram of a 67-year-old woman with haemoptysis; **C** a solitary PAVM was detected in the right middle lobe (arrow). **D** embolisation was performed with multiple coils (arrow)

Eighteen patients were treated for 28 lesions, and the success rate of embolotherapy was 100%. Four lesions (two lesions in the haemothorax group and two in the haemoptysis group) were embolised with plugs, and the remaining lesions were embolised with coils (Fig. 5). In the haemoptysis group, one patient received 2 units packed red blood cells before embolotherapy. Closed thoracic drainage was performed in four patients with haemothorax. No patients developed other complications during the peri-procedural period.

There were significant differences in the PaO2, SaO2, haemoglobin concentration, and leukocyte count before and after therapy in both the haemoptysis group (*P* = 0.004, *P* < 0.001, *P* = 0.009, and *P* = 0.048, respectively) and haemothorax group (*P* < 0.001, *P* < 0.02, *P* = 0.003, and *P* < 0.001, respectively) (Fig. 4B, C). The mean post-procedure hospital length of stay was 3.9 ± 1.5 days in the haemoptysis group and 6.4 ± 2.9 days in the haemothorax group.

In the haemoptysis group, three patients had previously undergone one session of ineffective bronchial arterial embolism (BAE) without pre-procedural CTA in other institutions. One patient with haemoptysis had undergone a session of ineffective BAE (neglected PAVM in our institution). In the haemothorax group, two patients were misdiagnosed as having lung cancer on non-contrast CT at another institution. One patient was misdiagnosed as having hydrothorax on non-contrast CT at another institution. One patient was misdiagnosed as having a myocardial infarction without a CT scan in the primary survey (Table 3).

**Table 3:** Laboratory indexes of patients with haemothorax in the first survey

Patient	ECG	PT(s)	D-Dimer (ng/ml)	Haemoglobin(g/L)	First diagnosis
1	N	12.3	707	95	Myocardial infarction
2	N	12.5	797	108	Malignant pleural effusion
3	N	11.2	2380	114	Haemothorax
4	N	13	550	100.4	Hydrothorax
5	Sinus tachycardia	11.7	2228	86	Malignant pleural effusion

*ECG* electrocardiograph, *PT* prothrombin time, *N* normal

No patient developed recurrence of symptoms by 1 week after therapy. The mean follow-up time was 3.2 ± 2.5 years (Fig. 4D). Two patients in the haemoptysis group died during follow-up. One patient with an acquired PAVM due to liver cirrhosis died of severe hepatic failure 8 months after embolotherapy. One patient died of heart failure 12 months after embolotherapy. No patients showed recanalization of PAVMs during follow-up.

## Discussion

To the best of our knowledge, this study is the largest series of ruptured PAVMs to date [8, 17, 18, 20, 21, 23]. In our study, the incidence of ruptured PAVMs was 5.5%, which is less than the previous reports [11, 23]. We reason that the frequent use of CT imaging increased diagnostic rate of PAVMs [9]. Besides, previous studies. were only focused on patients with HHT, and patients without HHT were not included. Although in our patients ruptured PAVMs happened with no clear triggers, some authors have reported that pregnancy and pulmonary hypertension could be risk factors for PAVMs rupture [26, 33, 34].

Twelve of 18 patients had HHT, and 58.3% of these 12 patients were women. In our study, the presence of HHT-associated PAVMs, especially in women, was a significant risk factor for the PAVM rupture. This is in line with previous studies [3, 6, 35]. Insufficient clinical records in some of our patients prevented us from comparing rupture rate in HHT vs. non-HHT PAVMs. Although the prevalence of PAVM rupture is probability low, we strongly recommend to consider PAVM rupture when managing patients with haemothorax or haemoptysis, especially patients with HHT and women with a family history of HHT [36–38].

In the present study, 8 of 18 patients (44.4%) were initially misdiagnosed or had undergone ineffective treatment. We believed that the cause of the misdiagnosis was the absence of a pre-procedural CTA. In the haemothorax group, the rate of misdiagnosis was up to 80% (4/5). The main clinical findings were non-specific respiratory distress and chest pain. Laboratory indexes were not specific enough to confirm the presence of haemothorax. Besides, failure of attaining a proper diagnosis of HHT-related PAVMs was also an important reason of misdiagnosis of ruptured PAVMs [39].

To our knowledge, previous studies did not describe in detail imaging manifestations of ruptured PAVMs. We observed that ruptured PAVMs exhibited a regular pattern on imaging. In the haemoptysis group, the adjacent consolidation or ground-glass opacity did assist in the identification of ruptured lesions. Massive patchy shadows may, however, obscure ruptured PAVMs. In the haemothorax group, imaging features such as the “anomalous bulge” sign and the “double shadow” sign were helpful for confirming PAVMs rupture.

**Fig. 6:**
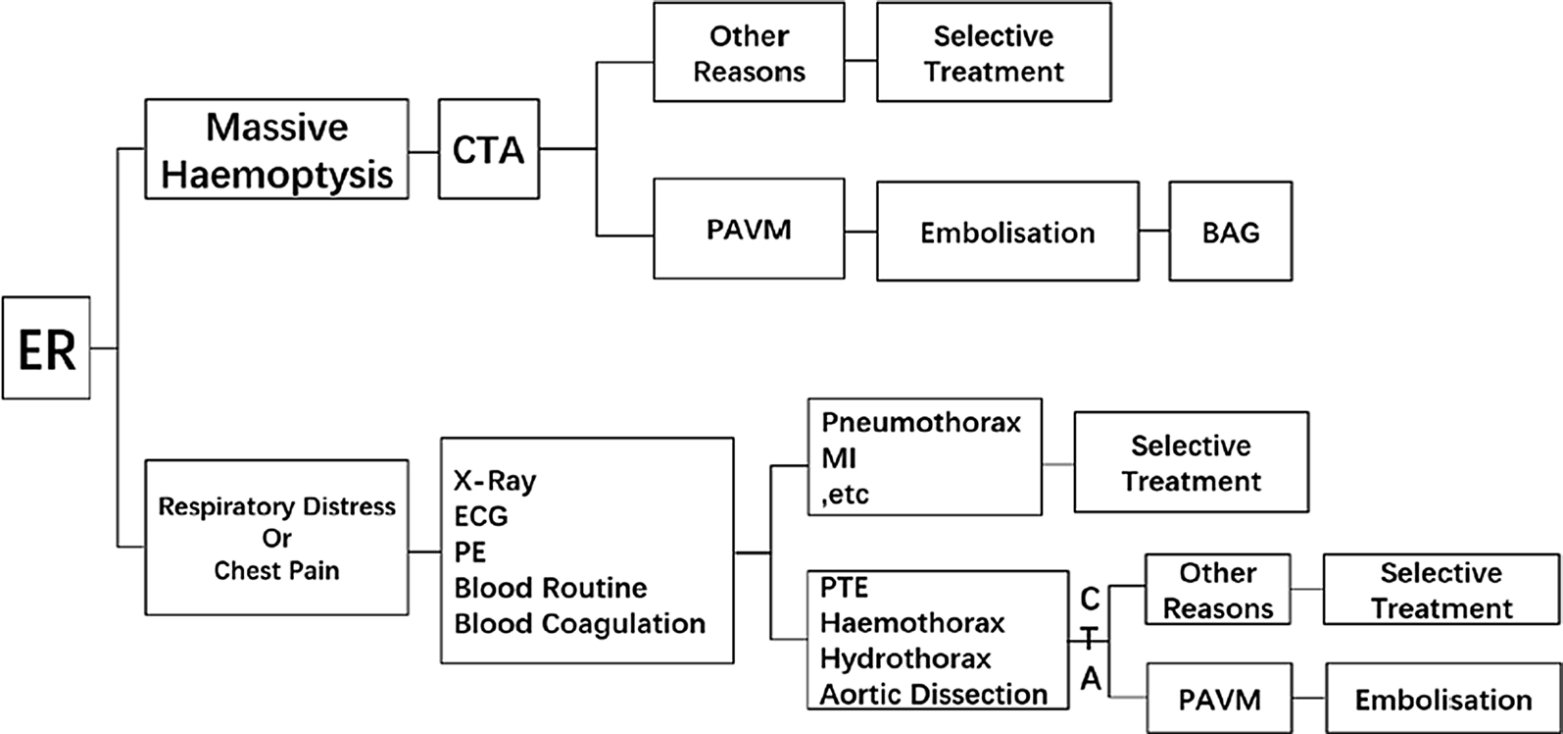
A clinical pathway is applied based upon the results of a patient assessment. Ruptured PAVMs usually presents as either massive haemoptysis or respiratory distress and chest pain. Patients with these clinical symptoms would be treated following the clinical pathway to detect the possibility of ruptured PAVMs. The clinical pathway we developed for our hospital has not been prospectively validated in other institutions. *ER* Emergency Room, *CTA* computed tomography angiography, *PAVM* Pulmonary arteriovenous malformation, *ECG* Electrocardiograph, *PE* Physical examination, *BAG* Bronchial arteriography, *PTE* Pulmonary thromboembolism

We observed that CTA can reveal afferent arteries, draining veins, and the sac of PAVMs. Moreover, volumetric reconstruction and maximum intensity projection are helpful in preoperative localization of ruptured PAVMs. This is why we consider pre-procedure CTA important as it effectively directs the next step of treatment [40, 41]. Even for patients in unstable conditions, we strongly recommended CTA after endotracheal intubation before therapy. Considering the active massive haemorrhage in this emergency condition, timely diagnosis and treatment are crucial. Based on our experience we developed a clinical pathway (Fig. 6). The intended goal of the pathway is to limit missing ruptured PAVMs in patients who present to the emergency department with haemoptysis, or respiratory distress and chest pain. However, this proposed pathway needs to be tested and validated in future study.

Treatment options of PAVMs include surgical resection, endovascular embolisation, and conservative medical treatment [24, 26, 40]. Patients with active massive haemorrhage have a high mortality rate during surgical resection because of haemorrhagic anemia, acute respiratory failure, hemodynamic instability. In addition, longer time is required to prepare for thoracoscopic surgery than for embolotherapy while the PAVM may continue to bleed or re-rupture. In contrast, embolotherapy may be more convenient and facilitate prompt haemostasis. Therefore, we recommended embolotherapy instead of thoracoscopic surgery for ruptured PAVMs [24, 26].

We propose that if multiple PAVMs were detected in one patient, other unruptured PAVMs (with feeding arteries ≥ 3 mm in diameter) should be embolised after embolisation of ruptured PAVMs. During the follow-up, different embolic materials, such as coils and plugs, presented the same therapeutic success [42].

We do not recommend chest drainage before embolisation in patients with haemothorax because intrathoracic decompression may worsen the haemorrhage or cause PAVM re-rupture [26]. We prefer closed thoracic drainage to thoracoscopic surgery after embolotherapy. Effective post-procedural drainage is good for recovery of patients with haemothorax. We observed mild haemorrhagic anemia and relatively low SaO2 in all patients before embolotherapy, and leukocytosis occurred 2 days after embolotherapy. Post-procedural administration of antibiotics and oxygen therapy may be helpful for recovery of patients with ruptured PAVMs.

Our study had some limitations. Because of the rare nature of ruptured PAVMs, our study was a small, retrospective single-center analysis, and comparation between embolisation and other treatment could not be performed. A family history of HHT was not confirmed in all 406 patients with PAVMs. We did not measure the pulmonary artery pressure before and after embolisation. Haemoglobin concentration could be a confounding factor, as being affected by potential extrapulmonary chronic bleeding in HHT patients.

## Conclusion

This study suggest that haemorrhagic complications caused by ruptured PAVMs are rare and often have no clear trigger yet, they can be life-threatening. HHT and the larger size of the afferent arteries seem to be important risk factors of PAVM rupture and haemorrhage. CTA is an ideal tool for diagnosis and guidance of management of ruptured PAVMs. We consider that timely embolotherapy led to our good clinical outcomes, and this regardless of embolic materials and techniques. Our results highlight the importance of a correct diagnosis and treatment strategy for ruptured PAVMs.

## Data Availability

The datasets used and analysed during the current study are available from the corresponding author on reasonable request.

## Abbreviations

PAVMs: Pulmonary arteriovenous malformations
CT: Computed tomography
CTA: Computed tomography angiography
HHT: Hereditary haemorrhagic telangiectasia
BAE: Bronchial arterial embolism
ECG: Electrocardiograph
ER: Emergency Room
PE: Physical examination
BAG: Bronchial arteriography
PTE: Pulmonary thromboembolism
PaO2: Partial pressure of oxygen
SaO2: Arterial oxygen saturation
